# Compensation after treatment for anterior cruciate ligament injuries: a review of compensation claims in Norway from 2005 to 2015

**DOI:** 10.1007/s00167-017-4809-y

**Published:** 2017-11-27

**Authors:** Per-Henrik Randsborg, Ida Rashida Kahn Bukholm, Rune Bruhn Jakobsen

**Affiliations:** 1 Department of Orthopaedic Surgery, Akershus University Hospital, 1478 Lørenskog, Norway; 2 The Norwegian System of Patient Injury Compensation, Postboks 3 St.Olavs plass, 0130 Oslo, Norway; 3 Department of Health Management and Health Economics, The Medical Faculty, University of Oslo, Oslo, Norway

**Keywords:** Anterior cruciate ligament, Patient compensation claim, Graft choice, Complication

## Abstract

**Purpose:**

To assess the most common reasons for complaints following anterior cruciate ligament (ACL) injuries reported to the Norwegian System of Patient Injury Compensation (NPE), and to view these complaints in light of the ACL reconstructions (ACL-Rs) reported to the Norwegian Knee Ligament Registry (NKLR).

**Method:**

Data from the NPE and the NKLR were collected for the study period (2005–2015). The age and gender and type of complaint and reason for granted compensation were collected from the NPE, while the graft choice and total number of ACL-R were collected from the NKLR. Risk for successful grant was estimated for graft type.

**Results:**

18,810 primary ACL-Rs were reported to the NKLR during the study period. A hamstring graft was used in 12,437 (66.1%) but the bone-patellar tendon-bone (BPTB) became the graft of choice at the end of the study period. 240 patients filed a complaint to the NPE, of which 101 were granted compensation. The odds ratio for a claim being granted following a hamstring graft was 2.9 compared to that of a BPTB graft (*p* = 0.002) The most common reason for compensation was a hospital-acquired infection in 39 patients (38.6%) followed by inadequate surgical technique (27, 26.7%) and delayed diagnosis (13, 12.9%). Of the 39 patients with infection, 27 had received a hamstring graft and six a BPTB graft (two patients were not reconstructed, data missing for three patients). Of the 27 patients who were granted compensation due to inadequate surgical technique, 24 had received a hamstring graft and three a BPTB graft.

**Conclusion:**

Infection and inadequate surgical technique are the most common causes for granted compensation from the NPE following ACL injury. Hamstring grafts have a threefold risk of complication that yields compensation from the NPE compared to BPTB grafts. This information is relevant for patients and surgeons when choosing graft type. The trend of increased use of BPTB grafts is warranted based on the results from this study.

**Level of evidence:**

Level III.

## Introduction

Rupture of the anterior cruciate ligament (ACL) is common and affects knee function with decreased ability to partake in sport activities. Reconstruction of the ACL is a frequently performed orthopedic procedure, with the aim to restore stability and allow the patient to return to an active lifestyle. ACL reconstruction (ACL-R) has a predictable good outcome, with the majority of patients achieving normal or nearly normal knee function [1]. Although complications are rare, graft failure, rotational instability and post-operative infections do occur, with potential detrimental results. To monitor the results after ACL-R, the Norwegian cruciate ligament registry (NCLR) was established in 2004 [7]. The NCLR provides a comprehensive overview of the nature of ACL-R taking place in Norway.

The Norwegian system of patient injury compensation (NPE) is a government agency handling compensation claims from patients who have suffered an injury in connection with the providing of health services, either within the public or the private healthcare sector. To be eligible for compensation the injury must have been caused by treatment, diagnosis, examination, caring or by lack of such. The injury must as a main rule been caused by failure in connection with the providing of health services, even if no one is to blame for this failure. Compensation may also be awarded even where no error or omission in treatment has occurred (e.g., when the injury is caused by technical failure of machinery, instruments or other equipment, by contagion or infection where this is not essentially caused by the patient’s condition or illness, by inoculation, or if the injury is particularly severe and unexpected). Furthermore, the injury must have caused a financial loss (e.g., of earnings and/or increased expenses). If the patient has not had a financial loss, compensation will not be granted. One exception to this rule is if the injury leads to a permanent medical impairment of at least 15%, in which case disability compensation may be awarded despite no financial loss. The degree of medical impairment is determined according to a preset table of injuries set in the regulations of the National Insurance Act where, for example, the loss of ACL is set to 5%. This might be the case if the patient is retired or can continue to work full time despite the disability. Finally, the patient must file a claim within 3 years from when it is reasonable to expect that the patient should realize that the injury is caused by the treatment or lack of treatment received. It is free to file a claim for compensation to the NPE, and the decision can be appealed at no cost for the patient.

The purpose of this study was to assess the most common reasons for complaints following the management of an ACL injury by evaluating the complaints filed to the NPE during the study period and compare the findings with the results from the NCLR in the same period.

## Materials and methods

Data from the NCLR were collected for the study period (2005–2015). The number of anterior cruciate ligament reconstructions performed in Norway was stratified by type of grafts used.

All complaints filed to the NPE following treatment for an anterior cruciate ligament injury in the study period were collected. The age and gender of the patient were collected, together with the type of treatment, graft choice, type of complication and any reoperations. The reasons for complaints and the compensation were reviewed, as well as the reasons for the non-granted compensation claims.

The study was approved by the data protection officer of Akershus University Hospital (study no 17-047). Approval from the regional ethical committee was deemed not necessary as all data were based on already anonymized records.

### Statistical analysis

The analysis was performed using IBM SPSS versus 22. Mean, median and standard deviation were calculated for continuous variables, and categorical data was presented in frequencies. Groups were compared using the two-sample independent *t* test or the Chi-square test. A *p* value < 0.05 was considered statistical significant. All tests were two-sided.

## Results

### Data from the NCLR

**Fig. 1:**
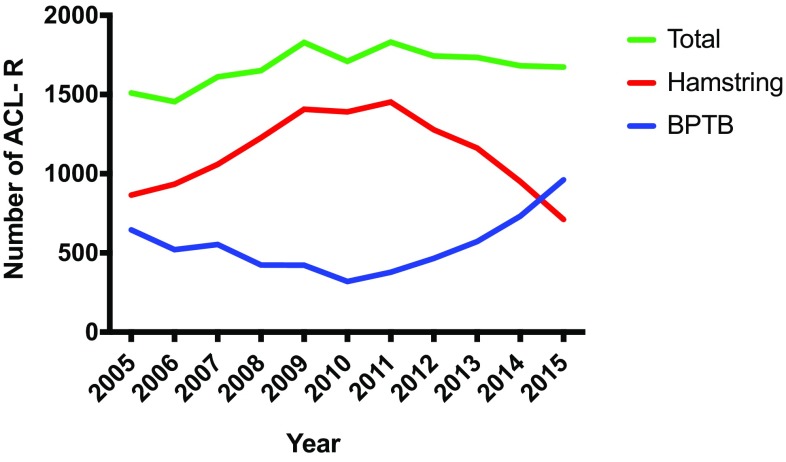
Primary ACL reconstructions (ACL-Rs) and graft choice in Norway between 2005 and 2015. *BPTB* bone-patellar tendon-bone

A total of 18,810 primary ACL-Rs were reported to the NCLR during the study period. A hamstring graft was used in 12,437 (66.1%) patients and a bone-patellar tendon-bone (BPTB) graft in 5993 (31.9%) patients, but the number of BPTB grafts increased at the end of the study period and is now the preferred graft in Norway (Fig. 1 **)**. Allografts are only rarely used in primary reconstructions in Norway, varying between 3 and 7 annually.

### Data from the NPE

**Fig. 2:**
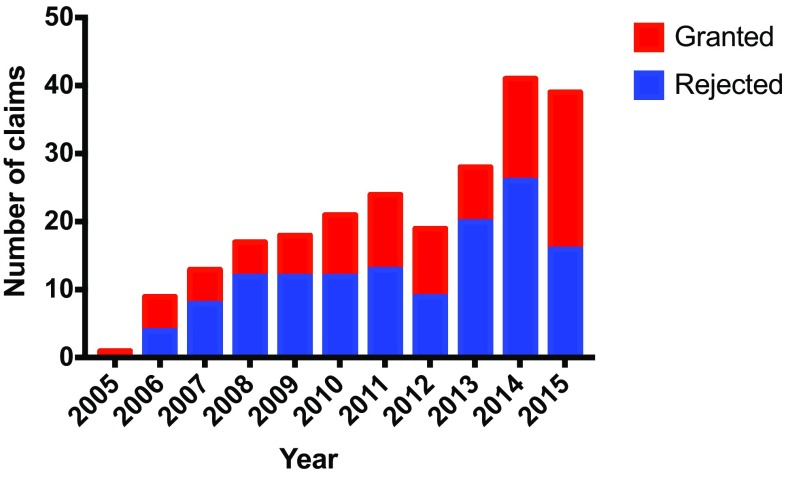
Complaints filed to the NPE between 2005 and 2015

During the study period there was a steady increase in claims for compensation while the number of primary ACL-R performed each year increased the first 6 years of the period and thereafter declined slightly. The mean percentage of granted claims was 47.4% with no obvious trend (Fig. 2). 240 claims were filed to the NPE during the study period. Compensation was granted to 99 patients initially, with another two claims granted after appeal, making the total number of granted compensation 101. The average age at the time of injury was 29.6 years (14–63). Men had a higher chance of being granted compensation (Table 1). Infection, instability, pain and stiffness were the most common reasons for complaints (Table 2), while infection was the most common cause for compensation followed by inadequate surgical technique and delayed diagnosis (Table 3). Of the 101 granted claims, 13 patients were treated without reconstruction (non-operatively or arthroscopically without ligament reconstruction). 67 patients received compensation due to a complication related to the surgery (such as infection or inadequate surgical technique), while 34 patients received compensation unrelated to the surgery, such as delayed diagnosis, anesthetic complications, and wrong-sided surgery. (Table 3). Of the 67 patients with surgery-related complications, 54 received a hamstring graft and nine patients received a BPTB graft (data missing in four patients). The odds ratio for a claim being granted following a hamstring graft was 2.9 (95% confidence interval 1.5–5.7) compared to that of a BPTB graft (*p* = 0.002) considering the overall graft choice in Norway during the study period. Of the 39 patients with infection, 27 had received a hamstring graft and six a BPTB graft, while three patients were not reconstructed (data missing in three patients). Of the 27 patients who were granted compensation due to inadequate surgical technique, 24 had received a hamstring graft and three a BPTB graft (*p* = 0.018). Wrong placement of graft was the major reason for inadequate surgical technique (21 of 27 patients) while four patients had metal (screws) protruding into the joint.

**Table 1:** Age and gender distributed by declined or granted claims

	Declined *n* = *139* (58%)	Granted, *n* = *101* (42%)	
Age, mean (SD, range)	31.6 (12.3) (14–75)	29.6 (10.4) (14–63)	N.S
Females, *n* (%)	78 (56.1)	41 (40.6)	*p* = 0.02

**Table 2:** Patients’ reasons for complaint in 101 patients granted compensation by the NPE

Reason for complaints (granted)	*N* = 101 (%)
Infection	26 (25.7)
Instability	23 (22.8)
Pain	20 (19.8)
Stiffness	11 (10.9)
Delayed diagnosis	7 (6.9)
Delayed treatment	5 (5.0)
Numbness	4 (4.0)
Wrong-side surgery	2 (2.0)
Fractured tooth during intubation	1 (1.0)
Wrong diagnosis	1 (1.0)
Data missing	1 (1.0)

**Table 3:** NPE reasons for compensation in 101 upheld claims

Reason for granted compensation	Number (%)
Hospital-acquired infection	39 (38.6)
Inadequate surgical tecnique	27 (26.7)
Delayed diagnosis	13 (12.9)
Surgery performed too late	6 (5.9)
Neurovascular injury	5 (5.0)
Wrong indication	2 (2.0)
Wrong side	2 (2.0)
Lack of documentation	2 (1.0)
Spinal infection	1 (1.0)
Spinal hematoma	1 (1.0)
Fractured tooth at intubation	1 (1.0)
Wrong primary care (casting)	1 (1.0)
Lack of follow-up	1 (1.0)
Total	101 (100)

The majority of claims was rejected by NPE because good medical practice was followed both in diagnosis and treatment (*n* = 101) or because no causal connection was found between complaint and treatment (*n* = 35). One claim was rejected due the patient being a smoker leaving him at higher risk for the pneumonia he acquired and two claims were rejected because there was no financial loss (Table 4).

**Table 4:** Patients’ reasons (summarized) for complaints in rejected claims

Reasons for complaint (rejected)	Number (%)
Pain or function deficit	61 (43.9)
Delayed diagnosis or treatment	46 (33.1)
Substandard care	16 (11.5)
Local infection, tissue or nerve injury	14 (10.1)
Deep vein thrombosis	2 (1.4)
Total	139

Complaints deriving from private hospitals (41/240) were compensated more often (23/101) than complaints from public hospitals (78/101) (*p* = 0.046).

A total of €2,436,000 [NOK 21,681,750 (1 € ≈ 8.9 NOK)] has been paid in compensation with an average payment of €24,200 (NOK 215,512). However, the median compensation was €3370, range €560–605,100 [NOK 30,000 (range 5000–5,385,723)]. The skewed distribution of compensation was largely caused by five extreme outliers with compensations over €110,000 (1 million NOK): Three patients received compensation between 1 and 2 million NOK. A 25-year-old female received €516,400 (NOK 4,596,570) due to permanent neurological injury following a spinal hematoma in relation to the anesthesia. The highest compensation of €605,100 (NOK 5,385,723) was awarded a 32-year-old male who sustained a permanent saphenous nerve deficit with subsequent pain, altered skin sensation and stiffness.

## Discussion

The most important finding of the present study was that the risk for a complication that leads to compensation by the NPE is nearly three times higher if a hamstring graft is used compared to a BPTB graft. This is in agreement with previous published data from the Norwegian ACL registry, demonstrating a twofold risk of revision surgery following Hamstring graft compared to BPTB graft [13]. The main reason for granted compensation following anterior cruciate ligament reconstruction is a hospital-acquired infection, followed by a misplaced graft and subsequent graft failure.

Hamstring grafts are popular, and preferred by many surgeons due to easy harvest, reduced post-operative pain and less anterior knee pain [4, 12]. The main argument to avoid BPTB grafts is more post-operative pain and anterior knee pain with the inability to kneel [16]. However, recent evidence from the ACL registries indicate that hamstring grafts have a higher risk of failure compared to BPTB grafts, especially in young and active patients [5, 14]. Patellar tendon grafts have therefore been recommended by some authors as the graft of choice in primary ACL reconstruction surgery [5]. The data from NCLR presented in the current study indicate that surgeons in Norway have acknowledged this evidence and BPTB grafts are now overtaking hamstring grafts as the graft of choice. This is in contrast to the report from Kaiser Permanente in the United States where graft choice remained stable from 2007 to 2014 [15].

Fortunately, infections following ACL-R are rare. 27 infected knees received a hamstring graft compared to only six patients who were infected following reconstruction with BPTB grafts. This difference did, however, not reach significance (*p* = 0.079), probably due to low numbers. However, our results are in accordance with previous studies that have found hamstring grafts to have a higher risk of infection compared to BPTB grafts [2, 3, 10]. The cause for this is unknown. The hamstring graft is a tendon deprived of its muscular attachment, while the BPTB graft is a true ligament with bony attachment at each end, perhaps increasing viability of the graft which protects against infection. Hamstring grafts takes longer to prepare [6], with whip sutures along the graft increasing the amount of foreign material in the joint, perhaps contributing to the increased risk of infection.

There was an increased risk of compensation due to inadequate surgical technique following hamstring grafts compared to BPTB graft. The most common error was wrong tunnel placement, and the most common complaint was instability due to graft failure. There is no reason to believe that tunnel placement is more difficult for hamstring grafts than BPTB grafts. However, it has been demonstrated that hamstring grafts have a higher mechanical failure rate, either by stretching of the graft or re-rupture [5, 14]. These patients are more likely to seek compensation via the NPE, and can explain the high number of hamstring cases with inadequate surgical technique found in our cohort.

The optimal timing of treatment is debated, but delayed diagnosis should be avoidable in a modern health care. Misreading of MRI or even the lack of reacting to a MRI results has caused compensation to be granted. Few patients were treated with immobilization, or without proper rehabilitation. Non-operative management is appropriate in some patients, but this involves a specific rehabilitation program [11]. If non-operative management is elected, proper follow-up is necessary to make sure intervention is carried out without undue delay if non-operative treatment fails. It is also worth mentioning that ligament reconstruction is not indicated in the absence of instability. One patient was granted compensation because non-operative management was not tried initially, and the surgery was deemed unnecessary because it was not clearly documented that the patient had instability prior to the surgery. Reflecting this, several claims, where patients seeked compensation because they felt diagnosis or surgery was delayed or initiated too late were rejected. The NPE stated that delayed surgery after physiotherapy and subsequent new clinical assessment is not substandard care, and thus compensation was not granted. This also reminds us that we do not treat the MRI results, but the patients’ subjective symptoms of instability, which should be reproducible by a positive pivot shift or a Lachman test without a firm end-point.

Three patients were granted compensation due to complications caused by the anesthesia. (One fractured tooth and one spinal infection and one spinal hematoma that needed surgical evacuation). Although this is not directly related to the surgical treatment per se, it reminds us that there is a general risk of complication with any type of operation, ligament reconstruction being no exception. A close collaboration between the anesthetic department and the surgeons are recommended to improve patient expectations and identify patients who might need special anesthetic attention or who are not suitable for the day surgery unit.

The median compensation from the NPE was €3370 indicating that the compensations paid after ACL-R are relatively minor. For comparison, a previous publication on NPE data found that the median compensation after peripheral nerve blocks was €39,000 (NOK 347,500). We also found that the highest compensation in our series was granted following neurovascular injuries with subsequent permanent disabilities. The average payment in our material of €24,200 is also considerably less than the average payment from NPE from all fields of medicine which was €65,400 (NOK 582,000) in the same period [9].

Two patients were operated on the wrong knee, which represent 0.01% of all ACL-Rs performed in Norway in the period. Although rare, this is an unacceptable and undefendable complication, and should not occur. The safe surgery checklist as proposed by the World Health Organization has been implemented in all public hospitals [8]. The two cases of wrong-sided surgery in this series were both performed in private hospitals, but whether safe surgery protocols were in use is not known.

This study is the first study analyzing patient reported claims in ACL reconstruction surgery in a national registry. This information may differ from the type of information that is obtained by the professional reported national ligament registries. Comparison of the two registries provides additional useful information about the nature and consequences of ligament reconstruction surgery. This insight into and awareness of patient experiences are important to improve patient safety.

It is important to emphasize that this is not a study on complications following ACL reconstruction, but an evaluation of the compensation claims received by the NPE following ACL injury. The NCLR has a reported registration rate of 86%, with no difference between public or private hospital [17]. Patients who are not in the registry can differ from the ones who are, and some patient with a complication might not have sought compensation via the NPE scheme. However, all public and most private hospitals in Norway take part in the NPE scheme, and it is unlikely that any potential missed cases would alter the general conclusions from this study. Unfortunately, NCLR do not publish data stratified by individual hospitals preventing us from using the NPE data to analyze relations between surgical volume or graft choice by institution.

The NPE does not comprise all complications following ACL surgery, and some patients might have suffered complications that would have led to compensation, but never filed a complaint to the NPE. The patients who did not file a compensation claim could differ from the patients who did, and this can represent a selection bias. Furthermore, only complications that cause a financial loss can be granted compensation. However, our results are generally similar to previously reported complications.

The assessment of each claim in NPE is also subject to several biases not readily controlled in a study like ours; for example, the decision to grant or reject a claim relies normally on one expert statement. To objectively study potential biases in the expert statements or the NPE procedures, we would need to assess this systematically for which we would have been obliged to seek consent from each of the 101 patients.

It is important to continuously search for areas of improvement in patient care. National registries such as the NCLR enable surgeons to learn from other people’s mistake. The present study highlights certain areas for potential improvement in the management of cruciate ligament injuries. Post-operative infection will always be a calculated risk following any surgical procedure. Attempting to reduce the infection rate by improved surgical techniques, adequate infection prophylaxis and graft choice should be an ongoing task. The surgical failures, such as misplacement of the graft, should be possible to avoid by improved training, proper supervision and possibly the use of preoperative fluoroscope to assure the correct position of the tunnels.

## Conclusions

Patient expectation management, sterile surgical conditions, proper graft handling and a thought through post-operative rehabilitation plan are keys to a successful ACL-R. BPTB grafts led to compensation due to infection and misplaced tunnels less frequently than hamstring grafts.
